# Song complexity is maintained during inter-population cultural transmission of humpback whale songs

**DOI:** 10.1038/s41598-022-12784-3

**Published:** 2022-05-30

**Authors:** Jenny A Allen, Ellen C. Garland, Claire Garrigue, Rebecca A. Dunlop, Michael J. Noad

**Affiliations:** 1 Cetacean Ecology Group, The University of Queensland, Moreton Bay Research Station, Dunwich, QLD 4183 Australia; 2 Southern Ocean Persistent Organic Pollutants Program, Centre for Planetary Health and Food Security, Griffith University, Gold Coast, QLD 4222 Australia; 3 School of Veterinary Science, The University of Queensland, QLD 4343 Gatton, Australia; 4 Centre for Social Learning and Cognitive Evolution & Sea Mammal Research Unit, School of Biology, University of St Andrews, St Andrews, Fife, KY16 8LB UK; 5 IRD, UMR ENTROPIE (Université de La Réunion, Université de la Nouvelle-Calédonie, CNRS, Ifremer, Laboratoire d’Excellence-CORAIL), BPA5 Nouméa, New Caledonia; 6 Opération Cétacés, Nouméa, New Caledonia

**Keywords:** Behavioural ecology, Evolutionary ecology, Population dynamics, Animal behaviour, Cultural evolution, Evolution of language

## Abstract

Among animal species, the songs of male humpback whales (*Megaptera novaeangliae*) are a rare example of social learning between entire populations. Understanding fine-scale similarity in song patterns and structural features will better clarify how accurately songs are learned during inter-population transmission. Here, six distinct song types (2009–2015) transmitted from the east Australian to New Caledonian populations were quantitatively analysed using fine-scale song features. Results found that New Caledonian whales learned each song type with high accuracy regardless of the pattern’s complexity. However, there were rare instances of themes (stereotyped patterns of sound units) only sung by a single population. These occurred more often in progressively changing ‘evolutionary’ songs compared to rapidly changing ‘revolutionary’ songs. Our results suggest that populations do not need to reduce complexity to accurately learn song patterns. Populations may also incorporate changes and embellishments into songs in the form of themes which are suggested to be learnt as distinct segments. Maintaining complex song patterns with such accuracy suggests significant acoustic contact, supporting the hypothesis that song learning may occur on shared feeding grounds or migration routes. This study improves the understanding of inter-population mechanisms for large-scale cultural transmission in animals.

## Introduction

Culture, once thought to be uniquely human, is found in a wide range of animal species. Individuals acquire a specific behaviour or trait through contact with another individual or their products, known as social learning^1^. Cultural transmission of these behaviours can occur between related individuals (e.g., tool use in a matriline of bottlenose dolphins [*Tursiops truncatus*]^2^), social groups (e.g., sweet potato washing in a tribe of Japanese macaques [*Macaca fuscata*]^3^), or populations (e.g., geographically distinct birdsong dialects^4^). Primates and cetaceans possess a varied and complex set of cultural traits, surpassed only by those found in humans^5,6^. Studies across these taxa groups allow for a comparative exploration of the common evolutionary drivers that lead to cultural traditions and their transmission^7^. Importantly, studies on cetaceans broaden the scope of such comparisons by including species from a non-primate lineage. One exemplary model species to contribute to the discussion of animal cultural exchange is the humpback whale (*Megaptera novaeangliae*) due to the presence of cultural traits in multiple aspects of their ecology. These include novel foraging strategies^8^, maternally directed site fidelity^9–11^, and a complex song display^12–14^.

A clear example of broad-scale cultural exchange among multiple populations is the transmission of humpback whale song within the South Pacific meta-population^14^. Song patterns are transmitted eastward from the west Australian population first to east Australia, then to New Caledonia, Tonga, and American Samoa, and then on to the Cook Islands, and French Polynesia^14^. However, the mechanism(s) for this transmission are not fully understood. Payne and Guinee^15^ proposed three possible vectors for song transmission between populations: (1) inter-population movement of individuals within a season, (2) inter-population movement of individuals between seasons, or (3) acoustic contact along migratory routes or feeding grounds shared among populations. The first vector, inter-population movement within a season (June-November which encompasses both the migratory and breeding seasons in the South Pacific) is relatively rare in the South Pacific region, though it has been documented^10,16,17^. More commonly reported are inter-population movements between seasons^10,18^, shared migratory routes^19,20^, and singing on the Antarctic feeding grounds^21^, presenting the second and third vectors as more plausible mechanisms of transmission for the South Pacific. However, not all populations necessarily use the same mechanisms of song exchange. Movement of entire song patterns across multiple populations, as observed in the South Pacific, has not yet been documented in any other location worldwide^14,22^, or any other species except humans.

Humpback whale song is a long, complex vocal display produced solely by males^13^. Individual sounds called ‘units’ are arranged in a sequence, which is termed a ‘phrase’^13,23^. Phrases are repeated multiple times to create a ‘theme’. Themes are then sung in a consistent order without repetition, creating a ‘song’. The song evolves over time through small, progressive changes, which all singers adopt through social learning^24^. These changes result in each year’s song containing a slightly different arrangement, known as a ‘song type’. In the east Australian population, small, progressive changes to songs (known as ‘evolutions’^14,24,25^) tend to increase the song pattern’s complexity (quantified using ‘complexity scores’^26^). For example, songs increase in duration, new themes are added, and a wider variety of units are used as songs evolve. Following these evolutionary changes, songs may also undergo a radical population-wide change known as a ‘revolution’, where a different song type introduced from the west Australian population entirely replaces the existing song^12^. Revolutionary songs tend to have lower complexity than the songs they replace, possibly as a result of limitations in learning such a large amount of novel material^26^.

Much of what is known about humpback whale song learning and fine-scale song structure comes from the songs of the east Australian population^12,14,27,28^. There is typically a consistent one-year delay in song transmission from east Australia to its closest neighbouring population, New Caledonia^14,29^. Although the same eastward one-year transmission in song also occurs from the west Australian population to east Australia, this is intermittent and has only been documented in ‘revolution’ years^30^. Songs are transmitted with strong song similarity on a broad scale (i.e., the sequences and occurrences of the themes present)^14^. Understanding fine-scale similarity (i.e., the sequences and occurrences of individual sound units within each theme) in song patterns and structural features will better clarify how accurately songs are learned during inter-population transmission.

Here, we analysed six separate song types, four revolutions and two evolutions, that were first recorded in the east Australian population and subsequently transmitted to the New Caledonian population the following year. This allowed for a direct and fine-scale quantitative comparison of song features using the same song patterns sung by two separate populations, both in situations where a known song is modified with moderate amounts of novel material (‘evolutionary’) and where the song is unknown and entirely novel (‘revolutionary’). Complexity scores were calculated using fine-scale song features including both the total and unique number of units, phrase and song duration, and individual theme complexity level. Identifying fine-scale commonalities and differences across populations will complement previous broad-scale works and improve our understanding of how inter-population song learning occurs.

## Results

### Song sharing between populations

**Figure 1:**
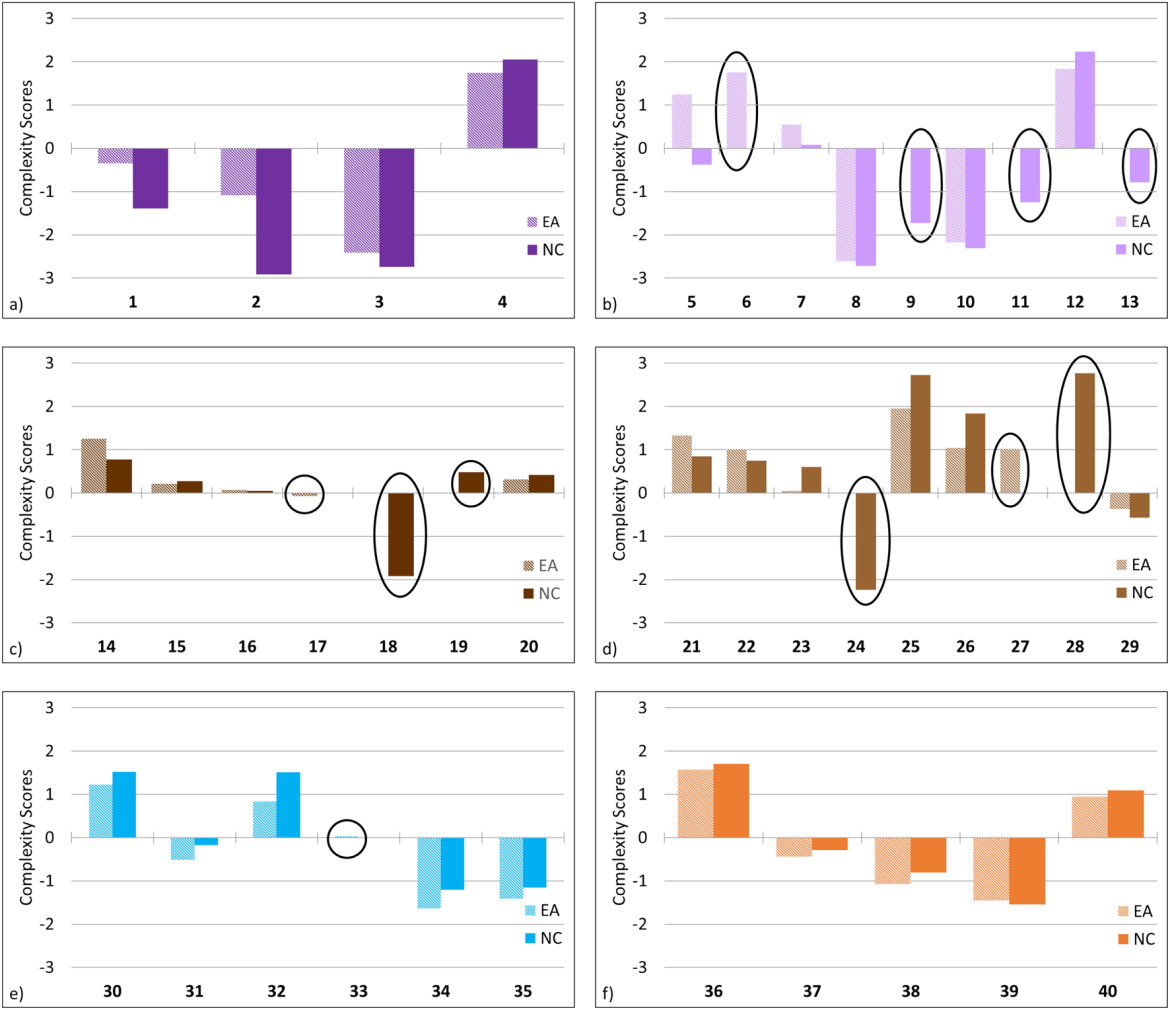
Complexity scores were calculated for every theme recorded for the (**a**) Purple song type, (**b**) Light Purple song type, (**c**) Brown song type, (**d**) Light Brown song type, (**e**) Teal song type, and (**f**) Orange song type. Scores were calculated for the song types as they appeared in each population. Themes are numbered, with unique themes that were only found in a single population circled in black.

Of the 40 total themes identified from the six song types (Purple, Light Purple, Brown, Light Brown, Teal, Orange, spectrograms in Figs. S1–6), most (29 themes) were shared between populations and a few (11 themes) were only recorded in a single population (Table 1). However, unique themes were rarely sung (across song types, unique themes only comprised 0–12.7% of all phrase repetitions, Figs. S1–6), precluding any consistent population-distinct versions of the respective song types. Unique themes occurred more frequently in the two evolutionary song types (Light Purple and Light Brown, Fig. 1) and were also more common in the New Caledonian population (Table 1). Neither shared nor unique themes had defining acoustic characteristics. Both types of themes contained units that covered the spectrum of acoustic features such as frequency (i.e., low- and high-pitched calls), duration (i.e., short and long calls), and modulation (i.e., flat and fluctuating calls). Such a range of call types suggests that acoustic features were not a driving factor in whether a theme was transmitted from one population to another.

**Table 1:**
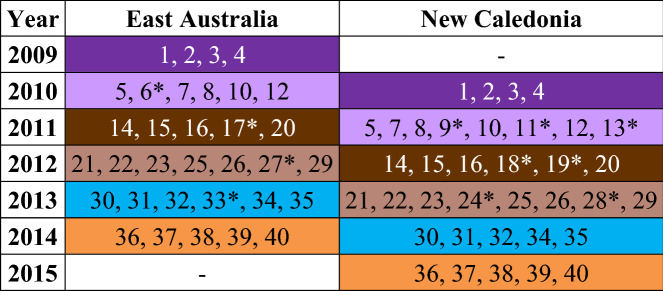
Song type and themes present in the east Australian and New Caledonian populations during the study.

Song types are identified following from Garland, et al.^14^ and Rekdahl^30^: Purple, Light Purple, Brown, Light Brown, Teal, and Orange. Each colour represents a distinct song type. Different shades of a colour (i.e., Purple and Light Purple) indicate related song types due to evolutionary song change. Revolution song types are Purple, Brown, Teal, and Orange. Evolution song types are Light Purple and Light Brown. Each distinct theme is numbered 1–40, with themes that were only found in a single population indicated with an asterisk (*).

### Complexity

**Figure 2:**
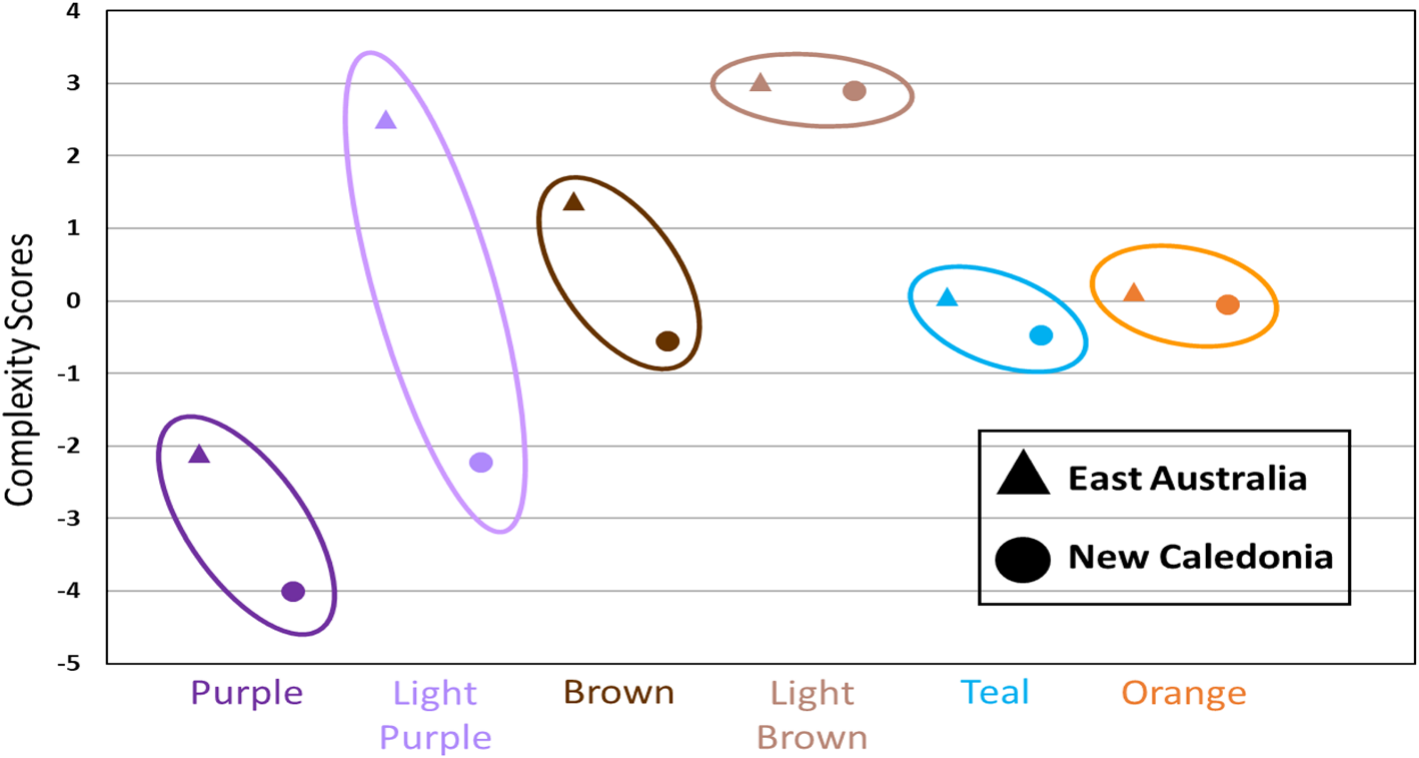
Complexity scores were calculated for each song type. Scores were calculated for each population using three variables at the song level (average total units, average unique units, average song length) and three variables at the theme level (average number of themes, average phrase length, average theme complexity).

Complexity scores were calculated using a Principal Component Analysis (PCA) of variables representing song pattern at both the song (average total units, average unique units, average song length) and theme (average number of themes, average phrase length, average theme complexity) levels of the song (Allen, et al.^26^). These scores serve as a reliable indicator of relative complexity within a song arrangement^26,31,32^. There were no significant differences in complexity scores of song types between songs recorded in the east Australian and songs recorded in the New Caledonian populations (Fig. 2, Mann–Whitney/Wilcoxon test, W = 28, *p* = 0.132). This indicates that if a song type was particularly simple or complex in the east Australian population, it remained so after transmission to New Caledonia. The only clear exception to this was the Light Purple song type, in which the complexity scores diverged sharply due to a single complex theme unique to the east Australian song (Theme 6), which was consistently present in all song cycles. Overall, complexity scores were lower in New Caledonia for all song types compared to east Australia. Further examination of the song sequences revealed that this was likely due to five of six east Australian song types having more total units per song cycle than New Caledonia. Despite these fine-scale differences, the overall song type was clearly retained between the east Australian and New Caledonia populations (see Methods: Verification of theme and song classifications).

There were no significant differences in the complexity scores of individual themes between populations for any song types (Fig. 1, Mann–Whitney/Wilcoxon, *p* > 0.4 for all song types). In most themes that were shared between populations, complexity was consistent relative to the other themes. In other words, themes that were simple (i.e., low complexity) in the east Australian population were also simple in the New Caledonian population. While shared themes had a range of complexity levels, themes that were unique to a single population tended to have consistently low complexity. The exceptions to this were Theme 6 from the Light Purple song type, and Theme 28 from the Light Brown song type (Fig. 1). Each of these themes had high complexity levels and were found in evolutionary song types.

## Discussion

Singers in New Caledonia learned the fine-scale unit arrangements of each song type with a high degree of fidelity. This demonstrates that song transmission from east Australia to New Caledonia is consistent at a fine-scale level of unit sequences as well as at the broad-scale level of theme sequences^14^. The high degrees of song similarity prevented analyses from distinguishing between populations, even with the occasional presence of themes unique to one population. High-fidelity copying was further supported by the comparable complexity levels for each song type and each of the shared themes between the populations. While east Australian song complexity was always higher, this difference was not significant and suggests that relative song complexity levels were largely maintained across song types. This was the case regardless of whether the song type itself was highly complex or not. Both the simplest song type (Purple) and the most complex song type (Light Brown) maintained similar complexity levels during transmission. The exception to these trends, found in the Light Purple song type, resulted from the presence of a common but single uniquely east Australian theme that had particularly high complexity (Fig. 1b), skewing the overall song type’s complexity level due to its consistent presence in all song cycles. Ultimately the lack of consistent differences in theme and unit arrangements demonstrates a lack of distinct “east Australian” or “New Caledonian” versions of each song type. Inter-population transmission therefore appears to be as robust as intra-population transmission, with similar degrees of within and between population variability. This strengthens the argument that song transmission is therefore likely to occur through close acoustic contact.

Themes that were shared by both populations contained units which covered the spectrum of acoustic features such as frequency, duration, and contour shape. Of note was the retention of sequences containing high frequency unit types (e.g., “whistles” or “squeaks”) during song transmission. Typically, high frequency signals do not propagate as far as low frequency signals and thus can be lost over distance^33,34^. Recent work by Girola, et al.^34^ also found that source levels (i.e., the sound level (dB re 1 μPa) at the acoustic source) in humpback whale song decrease with increasing frequency. The results found here highlight that the mechanism of song exchange between the populations allows for sufficient acoustic contact and high fidelity copying for all sequences to be learned (including those containing high frequency units). It further suggests that content-related factors such as novelty or learning ability might be more of a driving force in theme transmission as opposed to practical drivers such as an ability to hear certain elements of a theme.

The close acoustic contact could occur on the Antarctic feeding grounds^21^ or through the New Zealand migration corridor shared by both populations^19,20^. Recent work by Warren, et al.^35^ lends further strength to both of these as transmission mechanisms for these populations. Their detection of two song types on the Cook Strait migration corridor presents the possibility of song exchange with the required acoustic proximity suggested by our results. Additionally, song themes heard on east Australia’s southward migration were subsequently recorded in New Caledonia the following year, suggesting the additional exchange possibly of the shared Balleny Island feeding grounds. The limited individual exchange documented thus far between east Australia and New Caledonia^18^ suggests the movement of individuals either within or between seasons is a less likely mechanism for song transmission compared to feeding ground and/or migratory exchange, but cannot be ruled out. The one-year delay between the population is also not absolute, as Garland, et al.^14^ identified both populations singing the same song type at the same time in five separate years. When combined with the equivalent support for migratory and feeding ground transmission mechanisms, it is possible that there is flexibility in the transmission mechanisms used based on either ecological or environmental factors.

Themes that were unique to a specific population primarily occurred in evolving song types (Light Purple, Light Brown), which maintain part of their pattern from the previous year’s song. Incorporating larger sections of novel material (e.g., themes) might therefore only be attempted in songs with which singers are already at least partially familiar. Revolutionary song types are entirely novel material, which Allen, et al.^26^ suggest may be too demanding a learning task to allow for additional embellishments to be added at the time of learning. Given that the baseline learning requirements for an evolving song pattern is less or is built on prior learning, evolutions may either provide more opportunity to improvise/embellish or allow for copy error to occur more frequently as pattern complexity increases. The presence of population-specific themes suggests that these progressive song evolutions are indeed made through insertions and deletions at the theme level^36^, and spread among individuals within the population. Similar instances of population-specific themes have been previous identified across South Pacific populations, including both those in east Australia and New Caledonia^37^. These results support the proposed hypothesis that themes are the fundamental “building blocks” of song learning^38,39^. This corresponds with similar segmented learning mechanisms in other complex vocal communication systems such as bird song and human language. For example, some songbirds learn their song displays in smaller segments delineated by pauses^40^, while humans tend to use small components such as words or short phrases when learning language^41^. This further parallels other structural similarities between humpback whale song and both bird song and human communication, such as the underlying small-world structure found in all three taxa which is thought to facilitate learning^42^. A segmented learning mechanism may therefore be another key element to the cultural evolution and transmission of complex communication displays across taxa^38,43^.

More complex theme arrangements do not appear to be more difficult to learn, as both simple and complex shared themes retained their complexity levels during transmission with high levels of similarity (Figs. 1, S1–6). This corresponds with previous studies which have shown that more complex patterns do not contain more information and are likely to be embellishments or artifacts of the song learning process^26^. Such novel variations are thought to be driven by the changes of individual singers^44^, which are then learned by all singers through song conformity. However, many of the unit-level population differences in shared themes were substitutions of unit types with similar acoustic properties. Additionally, east Australia’s higher song-level complexity appears to be due to an increase in the total number of units present in the song rather than the number of unique units present. Rather than meaningful changes, it is more likely that these embellishments may be copy errors, flexibility in unit repetition, or unit types that singers may use interchangeably. Instead, a key source of novel material seems to be through the insertion of new themes, further supporting segmented learning.

These novel themes were disproportionately found in the New Caledonian population, suggesting that changes or embellishments of the song pattern were more common there. If complexity and novelty are indeed indications of learning capacity, as suggested in both songbirds^32,45,46^ and humpback whales^12,26^, then both maintaining complexity and adding novel material in a song arrangement are arguably more important in a breeding ground such as New Caledonia compared to the east Australian migratory corridor. More plausibly, the east Australian population may add their own novel themes when learning the song from west Australia. The New Caledonian population then learns the song with high fidelity (including most of east Australia’s added themes), and then attempts to incorporate their own novel themes as the song continues to evolve. If this is the case, then adding novel content in the form of these themes occurs during or after acquiring the new song. To test this, a fine-scale comparison of inter-population song transmission similar to those performed in this study should be conducted between the west and east Australian populations. More broadly, similar studies in other populations with the potential for acoustic contact (such as the Brazilian and Southwest African populations of the South Atlantic^47^) should be conducted to determine if the learning mechanisms identified here are species-wide.

Despite the prevalence of unique themes to New Caledonia, these unique themes tended to be simple while those unique to east Australia were often more complex. The much larger population in east Australia (~ 25,000^48^) has more singers and therefore may provide more sources for novelty for any given singer^44^. Conversely, New Caledonia (~ 1200^49^) has a smaller pool of individuals from which novelty may be drawn, as well as fewer individuals to spread the song among the population. Therefore, while novelty and embellishment could be more important in the breeding stock of New Caledonia, the small population size may restrict the complexity of the novel material that can be successfully introduced and incorporated into the population-wide song. This could also explain why east Australian songs as a whole tended to be more complex than New Caledonia, albeit not significantly. The role of these disparate population sizes is already hypothesized to drive the consistently eastward transmission of these songs across the South Pacific^22,50^, as the west Australian population is the largest and eastward populations get subsequently smaller^51^. Population size has further been shown to influence song learning in other species as well, such as the critically endangered honeyeater whose population decline is linked with a stark loss of vocal culture^52^.

## Conclusions

The consistent, directional song transmission between the east Australian and New Caledonian populations provides a unique opportunity to examine the product of song learning at an inter-population level. These populations are part of a broader case of horizontal cultural transmission on a large scale, the only example that has been documented in a non-human species^14^. We have quantitatively shown consistency in fine-scale structural features across populations to complement broad-scale analyses^14,22^. Complexity within these song types remained consistent during transmission, suggesting that constraints on song learning may not impact inter-population transmission. Although learning cannot be directly observed, song patterns provide indirect evidence regarding the mechanisms of song exchange in use. The results found here suggest a segmented learning mechanism with close acoustic contact (such as shared feeding grounds or migration routes), allowing for high fidelity copying of complex song arrangements between populations. Given that cultural exchange between populations is rarely documented in species other than humans, humpback whale song provides a model by which the evolution of cultural transmission in both animals and humans can be further understood.

## Methods

### Song collection and transcription

**Figure 3:**
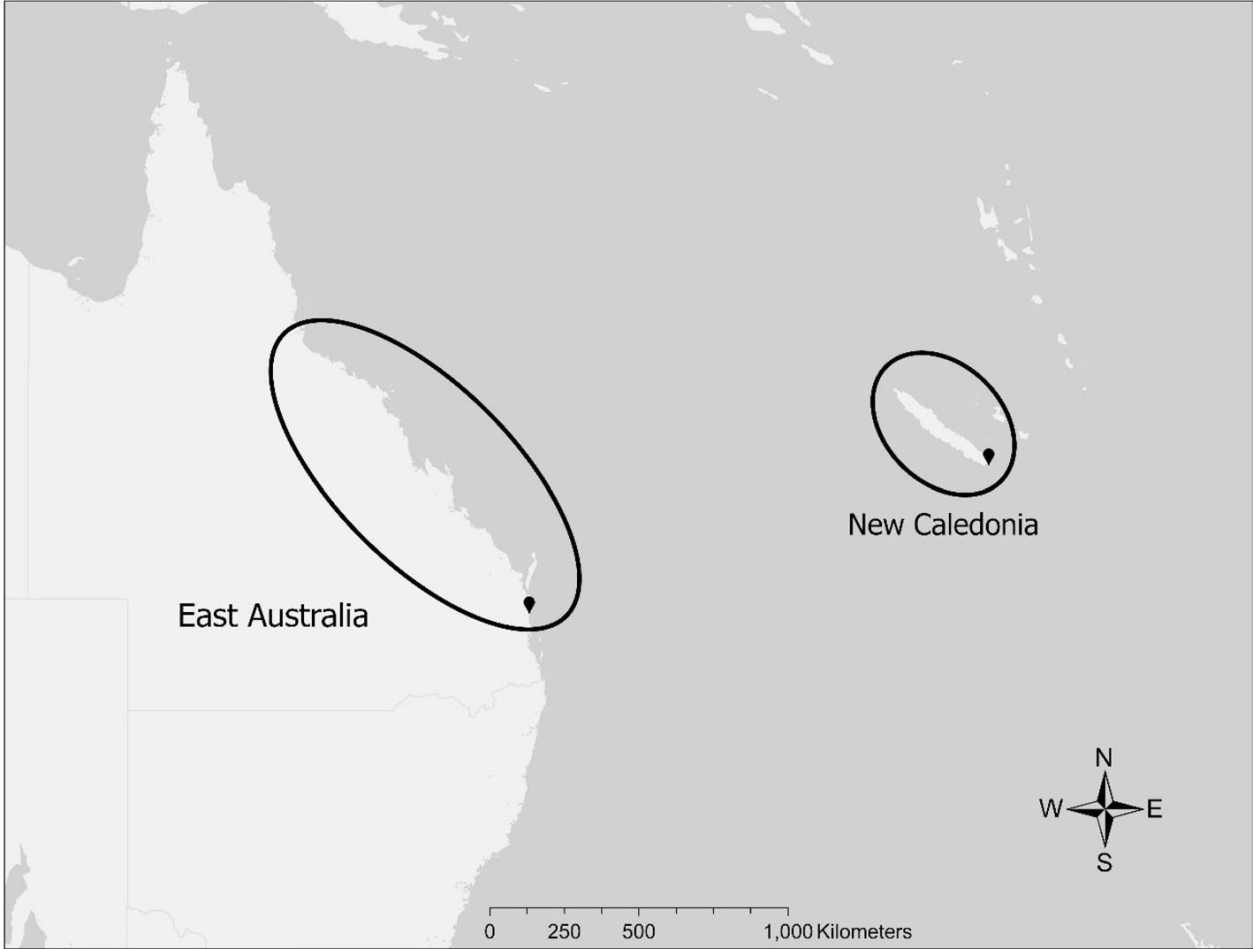
The location of each population, east Australia and New Caledonia, in relation to one another as well as deployment sites in black for data collection in each population.

Data were collected from two sites (Fig. 3): Peregian Beach on the coast of southeast Queensland, Australia (26°30’ S, 153°05’ E), and the southern lagoon of New Caledonia (22°28’ S, 166°56’ E). Passive acoustic recordings in east Australia were collected in 2009–2014 using two autonomous loggers (Acousonde 3A with external battery housings, Greenridge Sciences) and a fixed five-buoy hydrophone array (detailed in Allen, et al.^27^). New Caledonian recordings were made in the Southern Lagoon using boat-based hydrophones (2010–2013, HiTech) and Zoom recorder (16 bit, sampling rate of 44 kHz, WAV files), and a single passive acoustic recorder (2014–2015, SM2M + Whalesong recorder, Wildlife Acoustics with a sampling rate of 22 kHz). The years selected for analyses were based on the availability of high-quality recordings and resources for the subsequent transcription of recordings. All data were collected with approval from the University of Queensland Research and Innovation Animal Ethics (certificate approval numbers by year—2009: SVS/299/08/ACAMMS, 2010–2012: SVS/230/10/(NF), 2012–2013: SVS/403/12/EPSML. 2013–2014: CURTIN/SVS/283/1, 2014–2015: SVS/103/14). Data collection protocols were in accordance with relevant guidelines and regulations.

Spectrograms were generated in Raven Pro 1.6 (www.birds.cornell.edu/raven) and evaluated for quality following the methods outlined in Allen, et al.^27^. Song patterns were transcribed at the individual sound unit level using an acoustic dictionary developed from a subsample of measured units from each song type in each population (following Allen, et al.^27^). 353 complete song cycles (10–36 per year per population) were taken from 89 recordings, with each recording treated as a distinct singer following previous studies^42,53^. Each year song cycles were transcribed from a minimum of six individuals (excepting New Caledonia 2010, n = 4, due to a lack of high-quality recordings) which is considered to be sufficiently representative of the general population song pattern^14^. Although a small portion of singers in New Caledonia were recorded singing the 2013 song type in 2014, they were excluded from this study to focus on fine-scale comparisons between the same song pattern sung by the separate populations.

### Verification of theme and song classifications

Qualitative theme assignments were quantitatively verified for each population separately using the Levenshtein distance similarity index (LSI) analysis^26,28,54^. The calculation was weighted (β = 1) based on the acoustic feature similarity of units^26,27,55^. The LSI values were calculated between every pair of phrases (each phrase is composed of a sequence of units). The songs from each population in each year were first evaluated separately to identify the themes present and assign these to a song type (Figs. S1–6). The same song types from each population were then grouped together for comparison and assigned a colour name following previous work on these populations^14^ (Table 1). While transitional themes were eliminated due to their rarity and lack of stereotypy, all themes with at least two phrase repetitions in a song cycle were included.

This allowed themes to be assigned to each song type irrespective of population. Once themes had been verified, an unweighted LSI analysis was applied to the theme sequences of each song cycle to validate the qualitative song types (N = 10–36 song cycles per year per population, total N = 353 song cycles, Figs. S7 and 8). Theme sequences were used to account for the skewing that tends to occur in LSI analyses based on length^55^, following similar studies^14,37,56^. To identify population-level differences within each song type, separate similarity analyses were also conducted between every pair of song cycles per song type (Purple: N = 46, Light Purple: N = 54, Brown: N = 61, Light Brown: N = 48, Teal: N = 72, Orange: N = 72, Figs. S7 and 8. The inclusion of all song cycles ensured that both intra- and inter-individual variability could be accounted for^26^. The LSI matrix was clustered using average-linkage hierarchical clustering and bootstrapping using *pvclust* and *hclust* packages in *R*^57^ and custom written code (available at https://github.com/ellengarland/leven). Resulting dendrograms were assessed using the cophenetic correlation coefficient (CCC) to determine how well they represented associations within the data, with CCC > 0.8 indicating good representation^58^.

Based on their respective patterns, LSI grouped the song types (Purple, Light Purple, Brown, Light Brown, Teal, Orange) onto separate major dendrogram branches, and were clearly distinguished from one another (Figs. S7 and 8). On each major branch (i.e., each song type), the LSI further grouped each ‘song cycle’ (i.e., one set of specific themes sung in a stereotyped order^13,23^) regardless of the recording’s population of origin. Shared themes, which occurred in both populations, predominantly clustered together on the same branch (Figs. S1–6). This demonstrates that individual themes consistently retained their unit sequence patterns across populations in an accurate and recognisable way, providing quantitative evidence to confirm previous qualitative work^14^. Dendrograms for all song types had a cophenetic correlation coefficient (CCC) score of  > 0.9, indicating a good representation of the associations in the data.

### Complexity scores

Song complexity was evaluated using ‘complexity scores’ generated for each year’s song type following the methods presented in Allen, et al.^26^, modified from Boogert, et al.^32^ and Templeton, et al.^31^. Complexity scores were generated by reducing positively correlated variables to a single principal component using principal component analysis (PCA) with the *princomp* function in R (Version 4.0.5^59^). *Song* level variables represented the full sequence of units in the song cycle, including all phrase repetitions, using the following variables: (1) number of units per song cycle, (2) number of unit types per song cycle, and (3) duration of each song cycle (s). *Theme* level variables accounted for the presence of separate themes using the following variables: (1) number of themes per song cycle, (2) mean phrase duration per song cycle (s), and (3) mean individual theme complexity score per song cycle (calculated following Allen, et al.^26^). For each song type, these six variables were combined and condensed into a single composite score by the PCA, with this score represented by the first principal component score value. Scores have a direct, positive relationship with complexity: higher scores indicate higher relative complexity within the song’s pattern. To further evaluate population-level differences, a set of complexity scores was also calculated for each individual theme per population. Differences in separate theme complexities between the two populations were then evaluated using a non-parametric Mann–Whitney/Wilcoxon test. Statistical analyses were run in *R* (Version 4.0.5^59^).

## Supplementary Information

Supplementary Information 1.

Supplementary Information 2.

## Data Availability

All datasets generated during and/or analysed during this study are available on the Dryad Digital Repository [doi: 10.5061/dryad.9p8cz8wk1]. Please seek prior written permission from JAA to reuse data in any form except to confirm the results of this study.
