# Supplementary material for: Song complexity is maintained during inter-population cultural transmission of humpback whale songs
Source: Sci Rep. 2022 May 30;12:8999. doi: 10.1038/s41598-022-12784-3 (PMC9151787; doi:10.1038/s41598-022-12784-3)
Supplement: Supplementary file 1 — Supplementary Information 1. [file 41598_2022_12784_MOESM1_ESM.pdf]

Supplemental Figures S1-6 These figures show the features of each song type in both populations. Themes are all numbered 1-40 with an asterisk (*) to indicate a theme unique to a single population.

Supplementary Figures a-b Each figures represents a) a spectrogram of the east Australian song, b) a spectrogram of the New Caledonian song. Similarity Index for all themes and d) the relative occurrence of each theme. Spectrograms show a representative phrase for each theme.

Supplementary Figure c Dendrograms showing similarity in song structure across populations for each theme. Bootstrapped (1000 times) average linkage hierarchical cluster analysis was used based on similarity matrices of set median unit sequences of themes. The height (y-axis) of two branches reflects the LSI distance between them. Red dots indicate divisions with an approximately unbiased p-value greater than 95% and are thus strongly supported by the data, with red boxes around the resulting major branches. Themes are labelled by population (EA or NC) and theme number (1-40).

Supplementary Figure d The relative occurrence of each theme across populations (representation as a proportion).

Figure S1a-d The Purple song type from a) east Australia 2009 and b) New Caledonia 2010

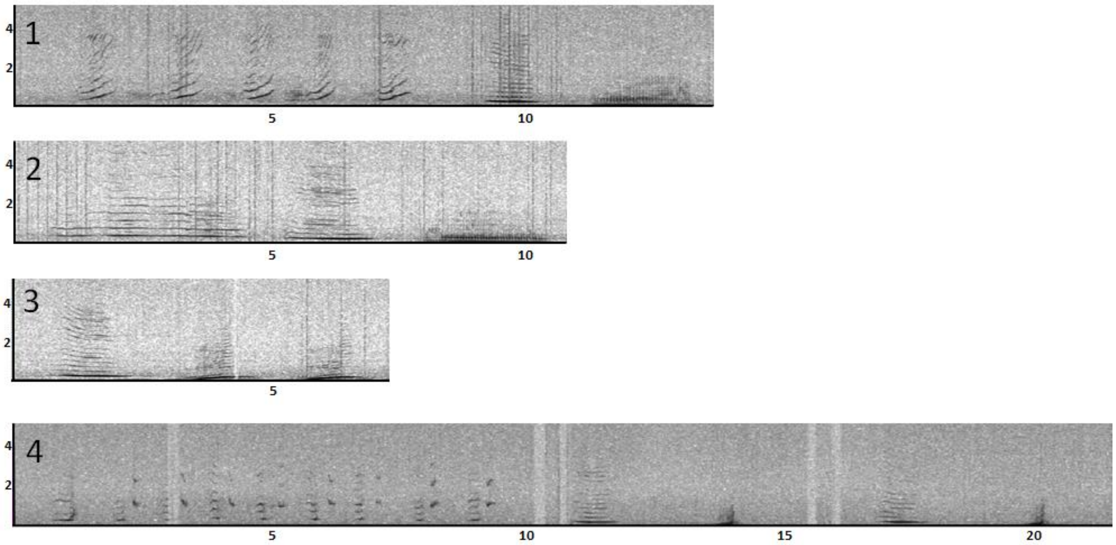

a)

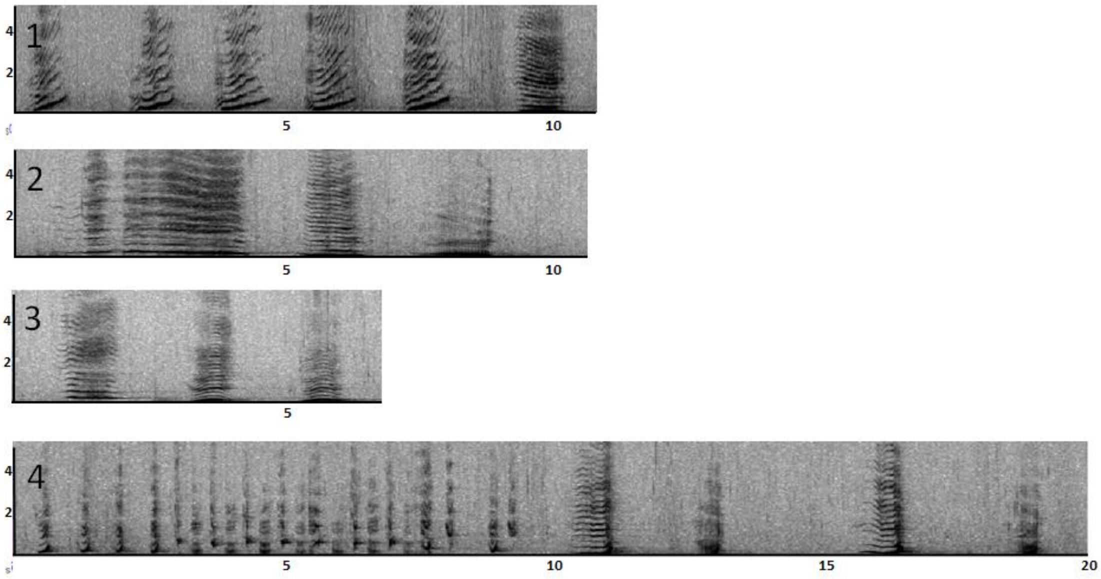

b)

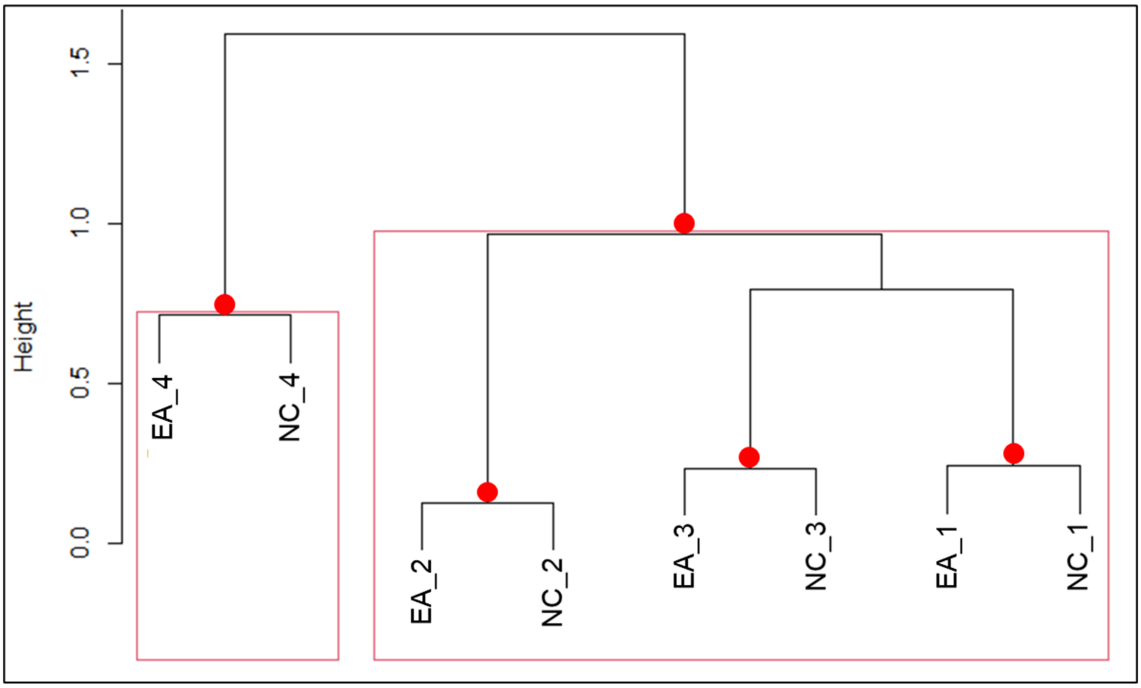
Height
1.5
1.0
0.5
0.0
EA_4
NC_4
EA_2
NC_2
EA_3
NC_3
EA_1
NC_1
c)

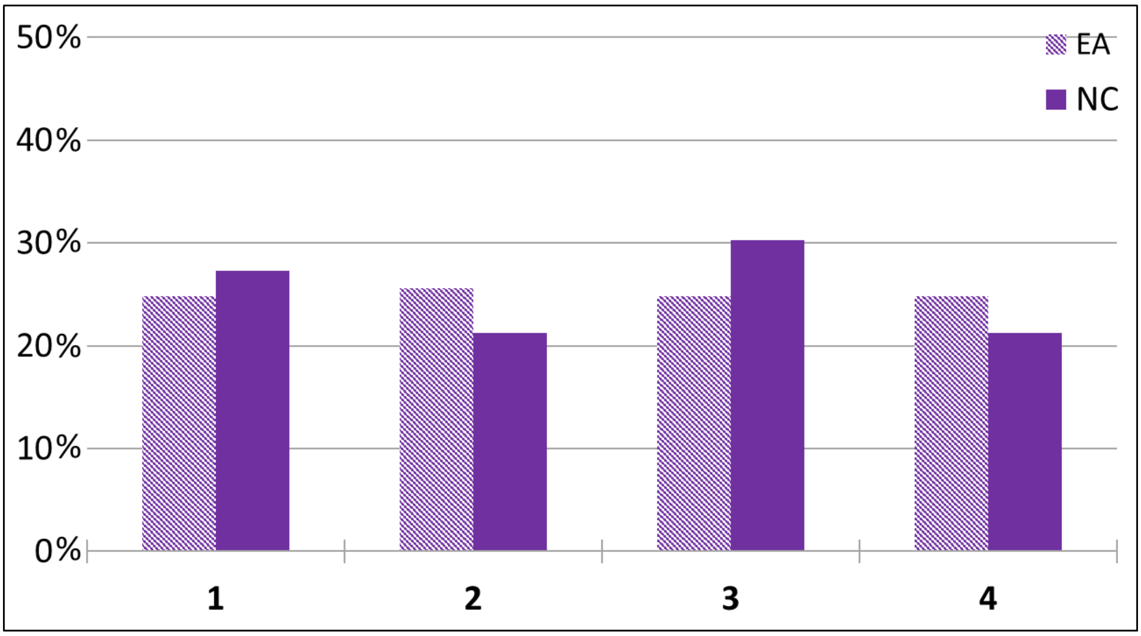
50%
40%
30%
20%
10%
0%
EA
NC
1
2
3
4
d)

Figure S2a-d The Light Purple song type from a) east Australia 2010 and b) New Caledonia 2011. Theme 5 evolved from Theme 1, Theme 7 evolved from Theme 2, and Theme 10 evolved from Theme 3.

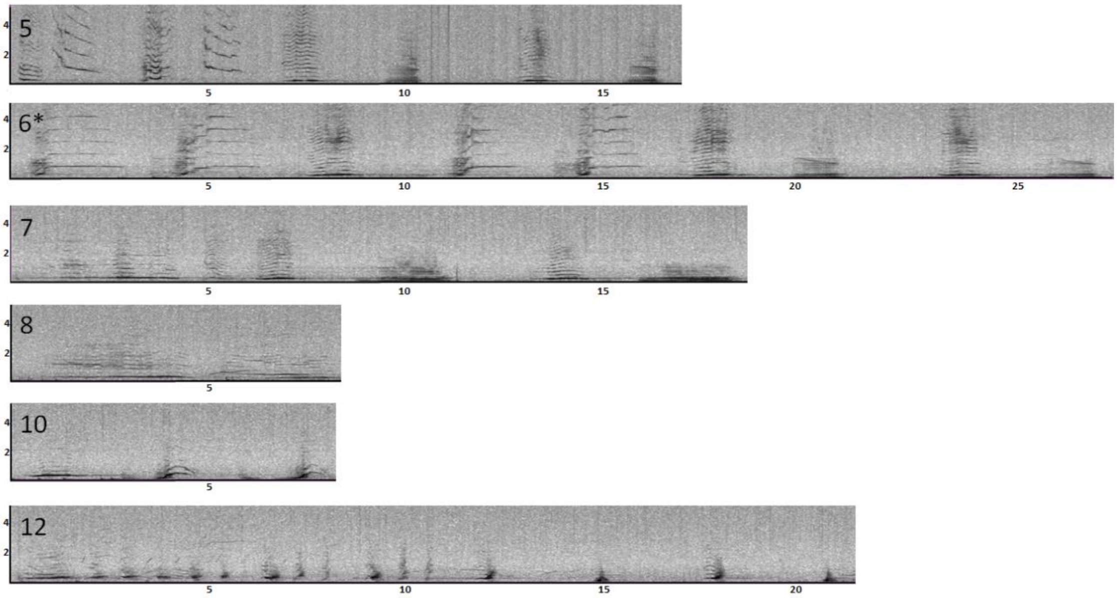

a)

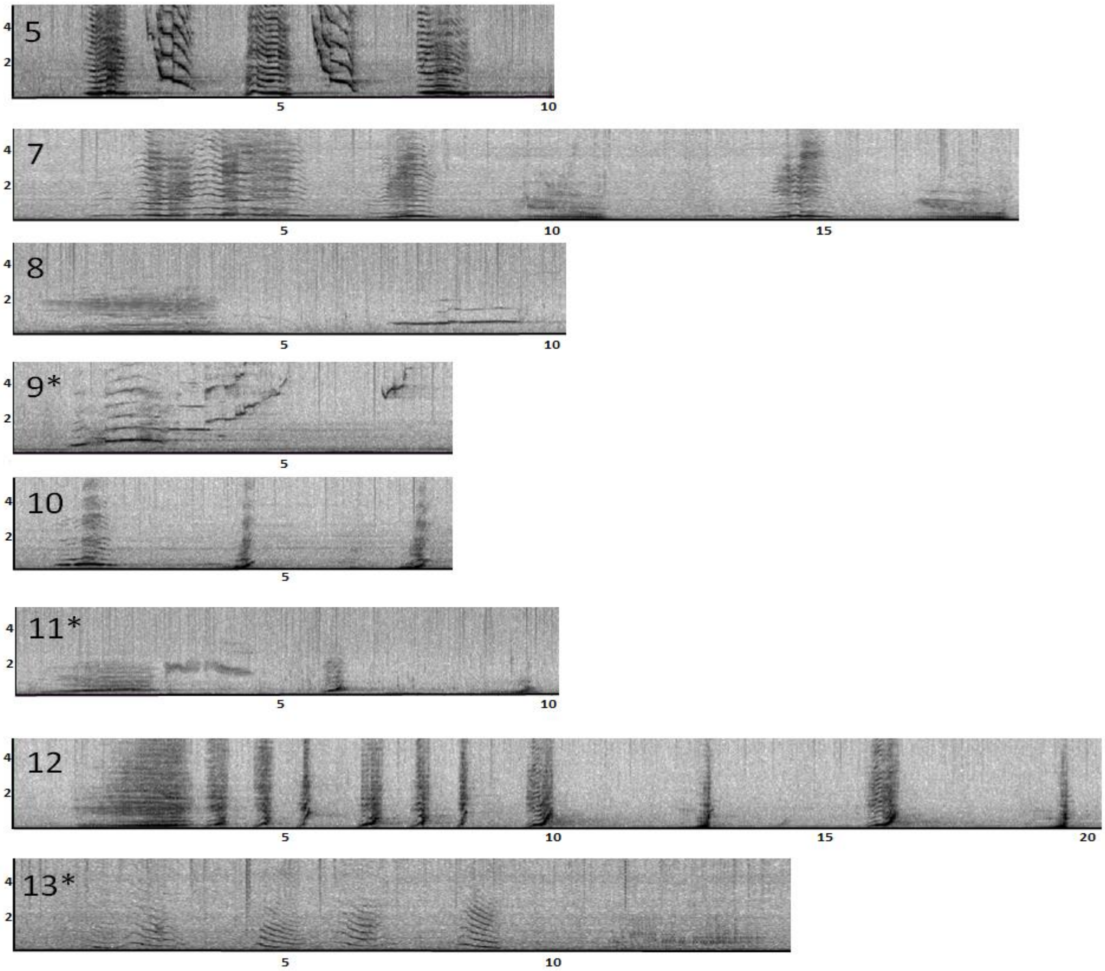

b)

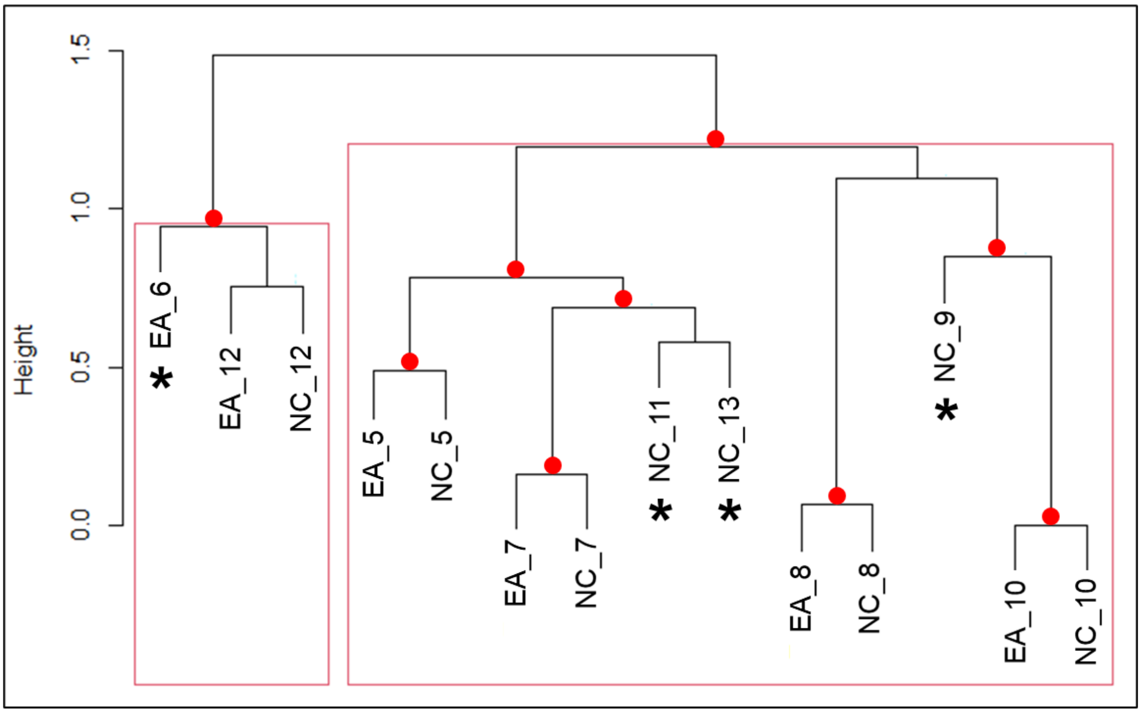
Height
1.5
1.0
0.5
0.0
EA_6
EA_12
NC_12
EA_5
NC_5
EA_7
NC_7
NC_11
NC_13
EA_8
NC_8
NC_9
EA_10
NC_10

c)

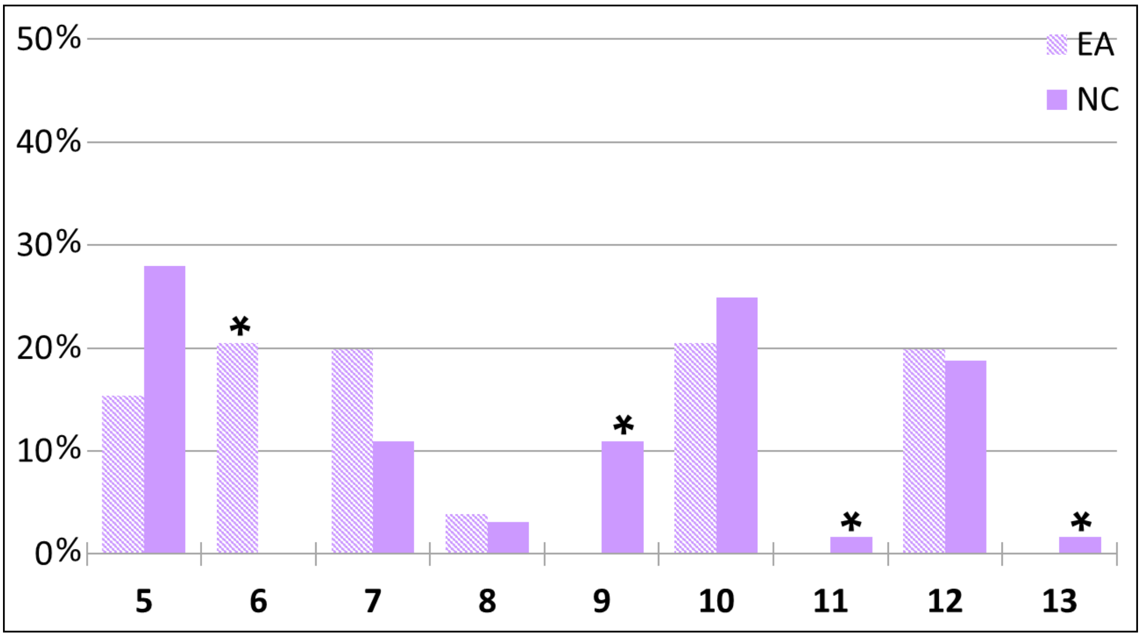
50%
40%
30%
20%
10%
0%
EA
NC
5
6
7
8
9
10
11
12
13

d)

Figure S3a-d The Brown song type from a) east Australia 2011 and b) New Caledonia 2012

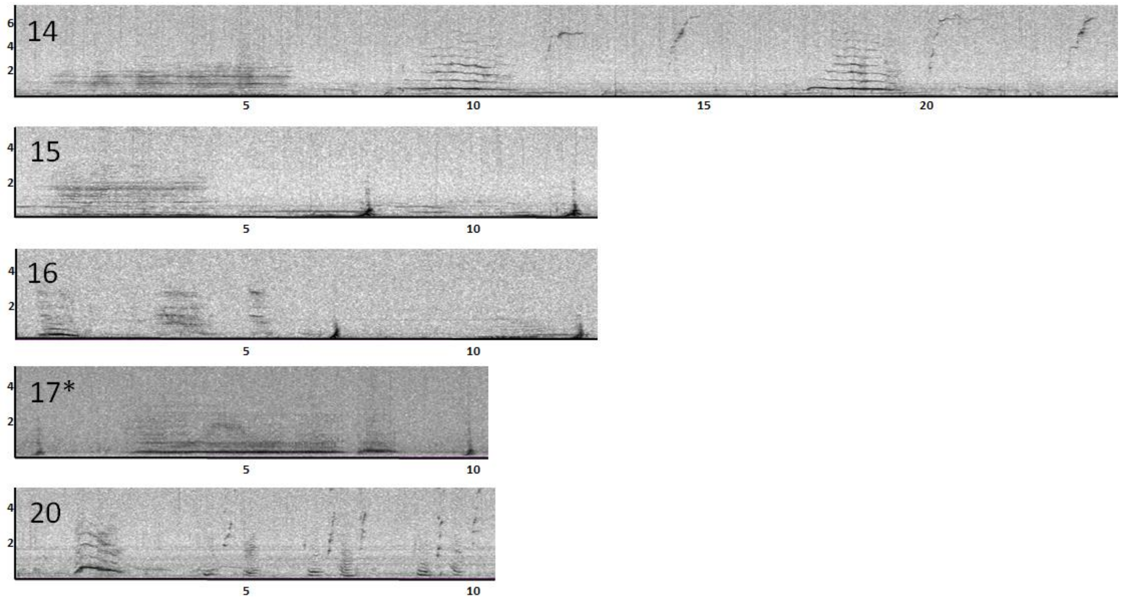

a)

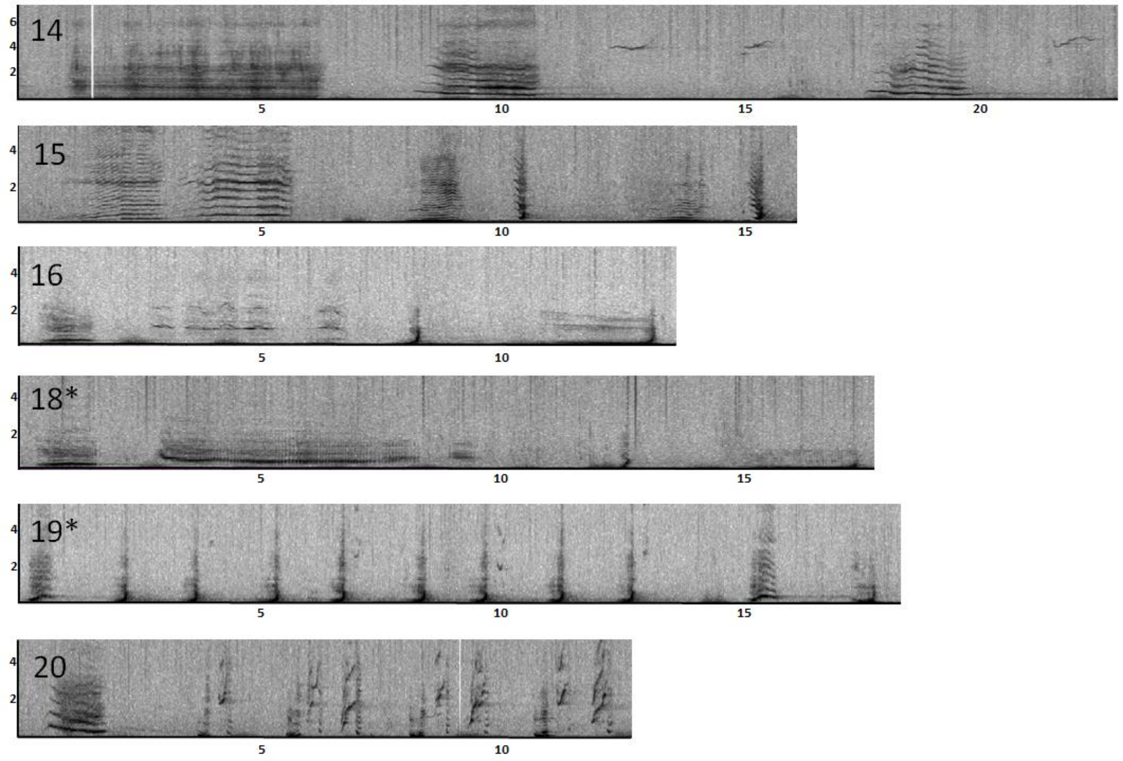

b)

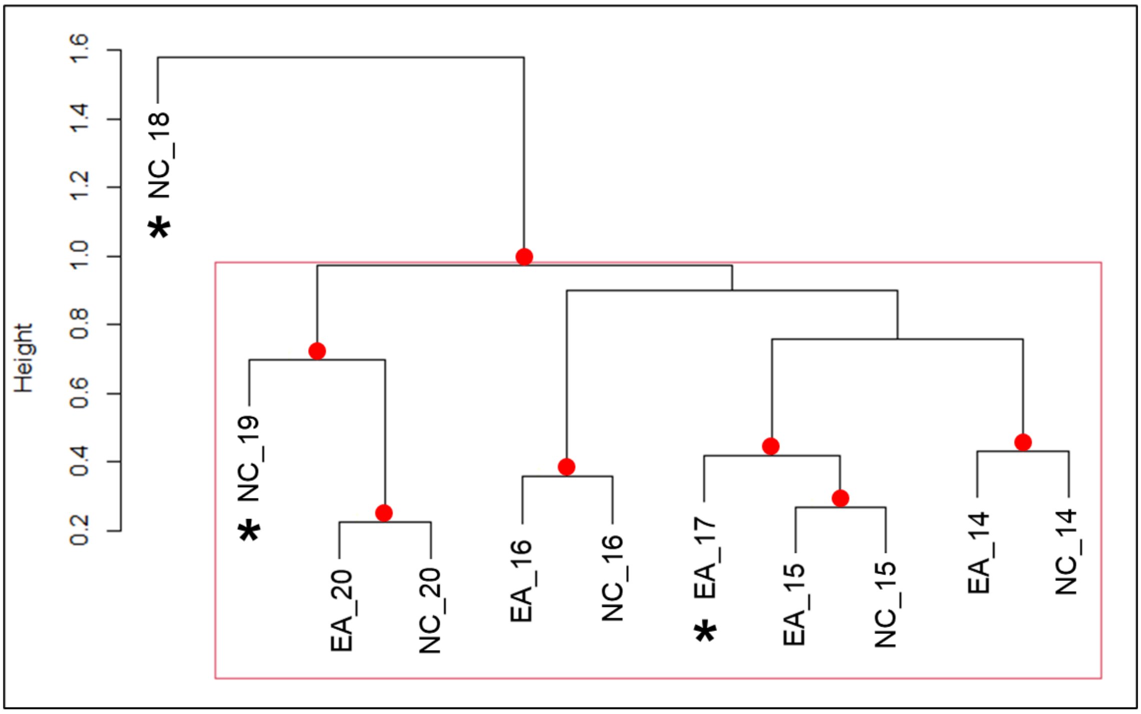
Height
1.6
1.4
1.2
1.0
0.8
0.6
0.4
0.2
* NC_18
* NC_19
EA_20
NC_20
EA_16
NC_16
* EA_17
EA_15
NC_15
EA_14
NC_14

c)

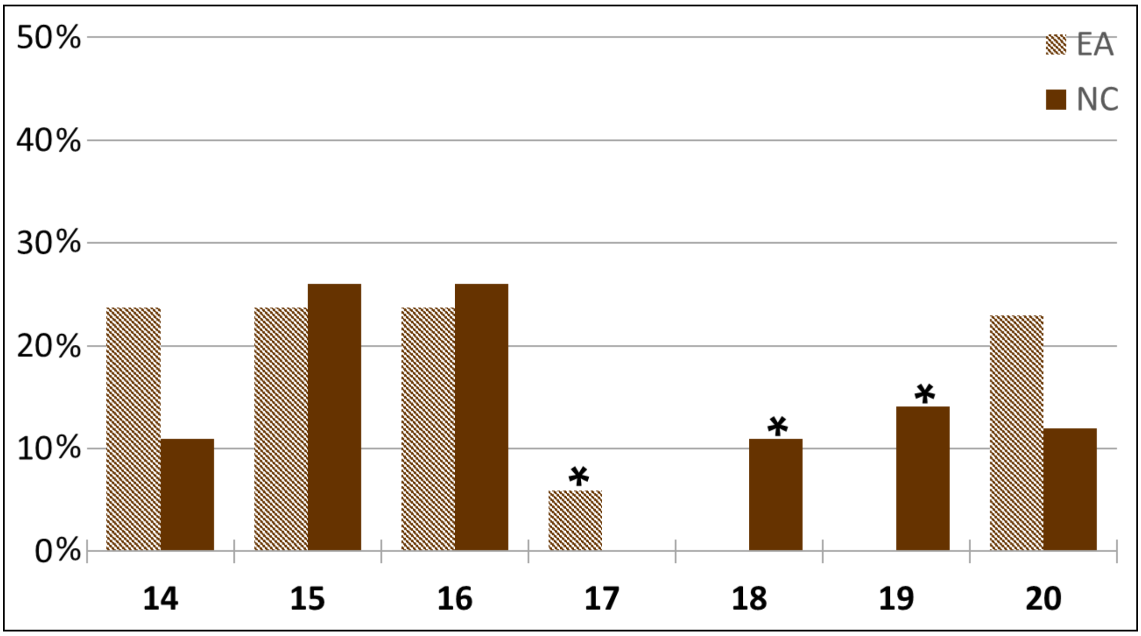
50%
40%
30%
20%
10%
0%
EA
NC
14
15
16
17
18
19
20

d)

Figure S4a-d The Light Brown song type from a) east Australia 2012 and b) New Caledonia 2013. Theme 21 evolved from Theme 14 and Theme 23 evolved from Theme 16.

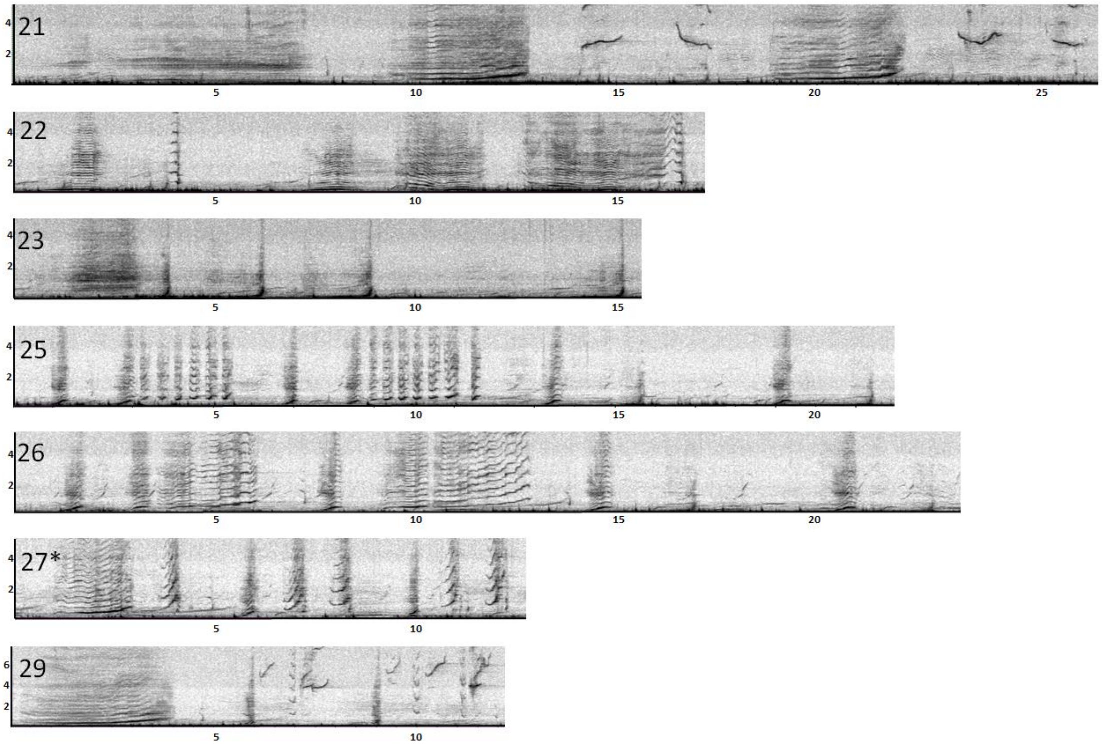

a)

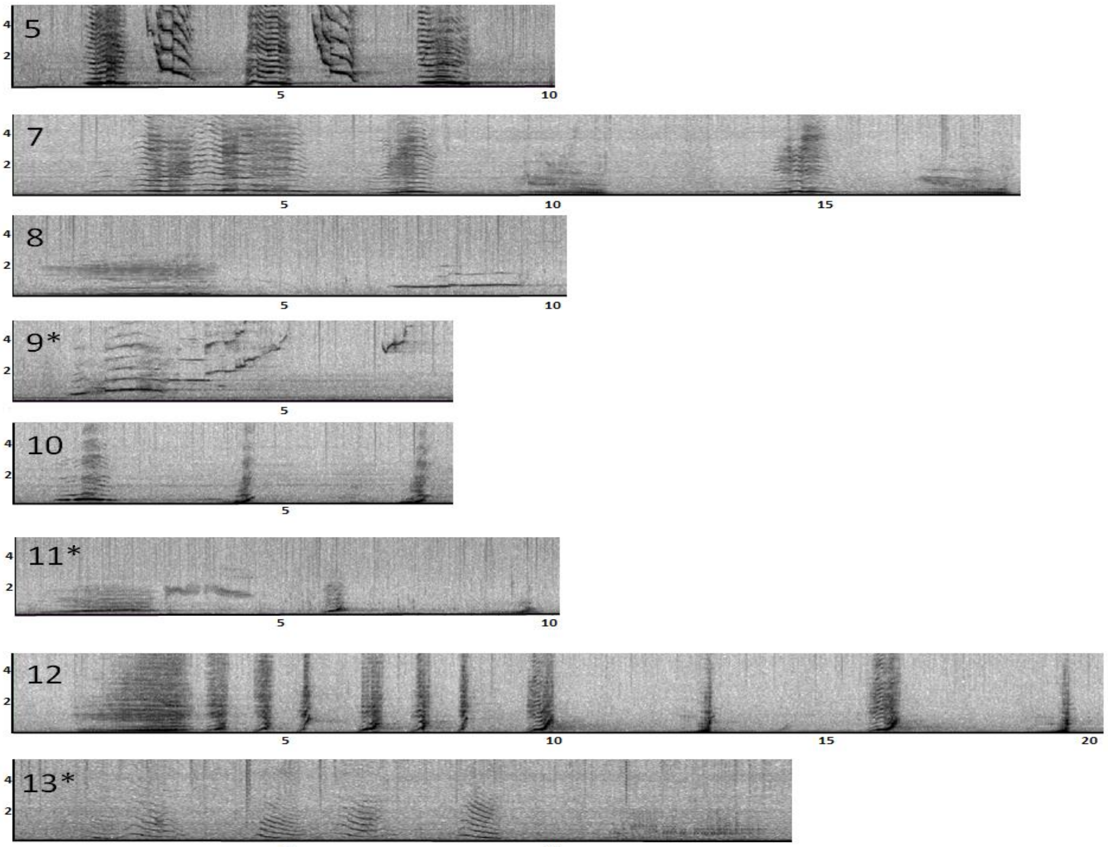

b)

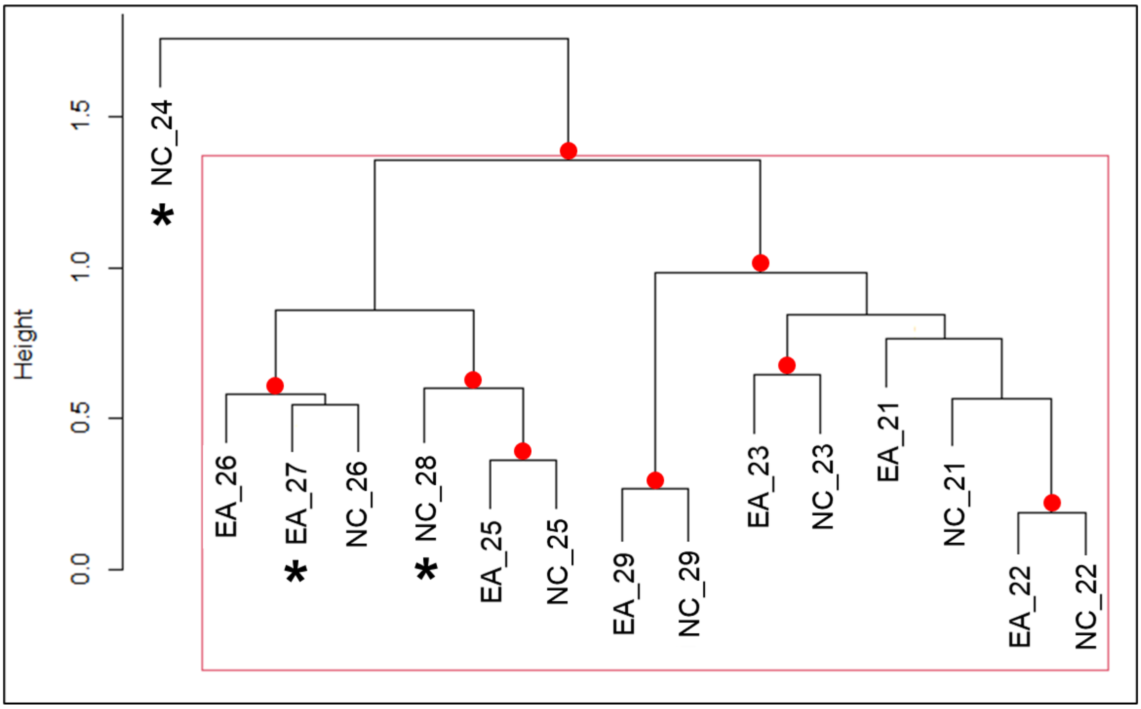
Height
0.0
0.5
1.0
1.5
* NC_24
EA_26
* EA_27
NC_26
* NC_28
EA_25
NC_25
EA_29
NC_29
EA_23
NC_23
EA_21
NC_21
EA_22
NC_22
c)

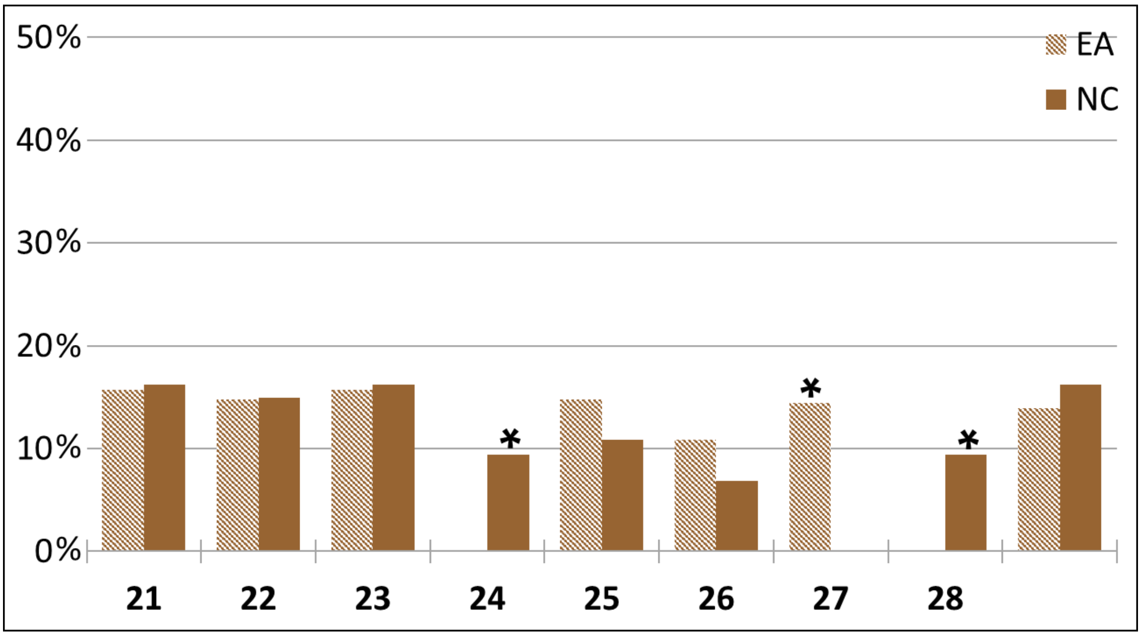
50%
40%
30%
20%
10%
0%
EA
NC
*
*
*
21
22
23
24
25
26
27
28
d)

Figure S5a-d The Teal song type from a) east Australia 2013 and b) New Caledonia 2014

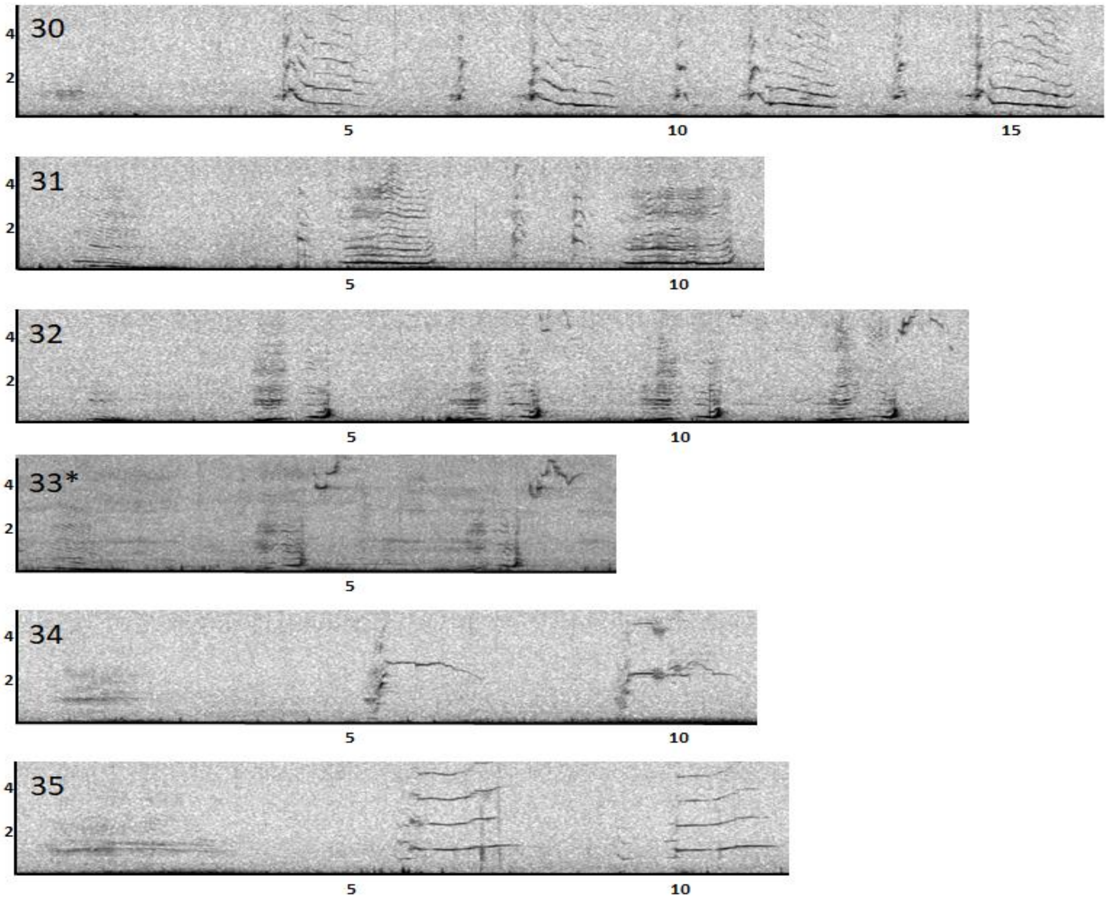

a)

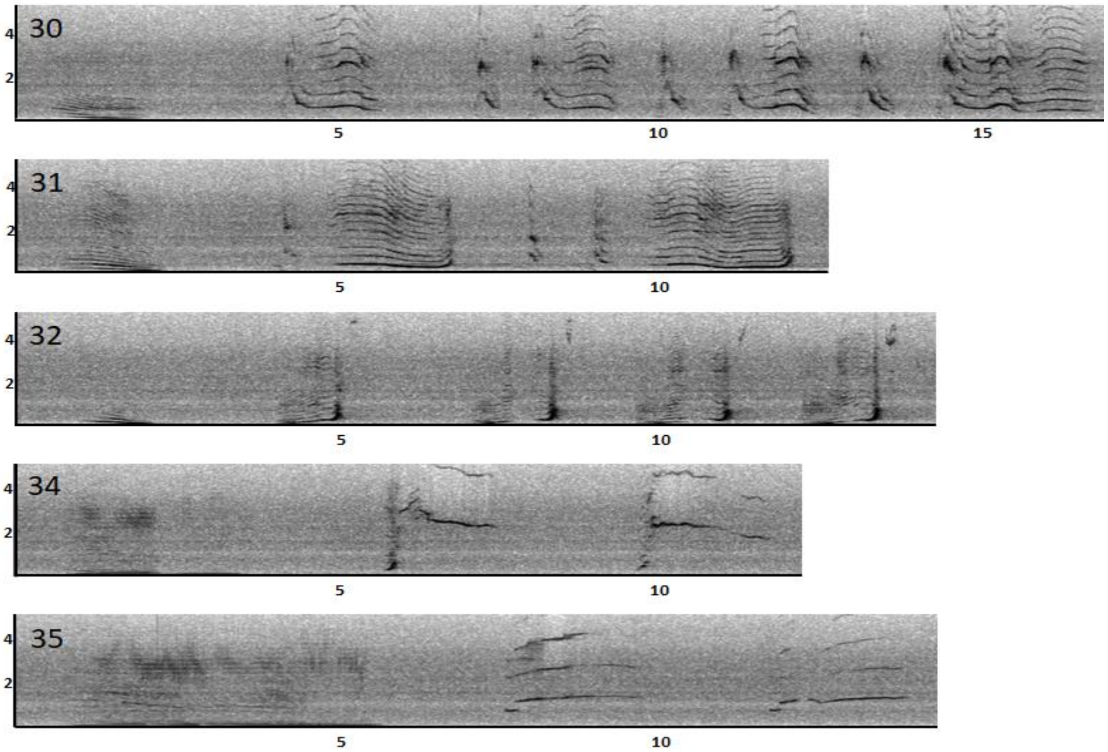

b)

c)

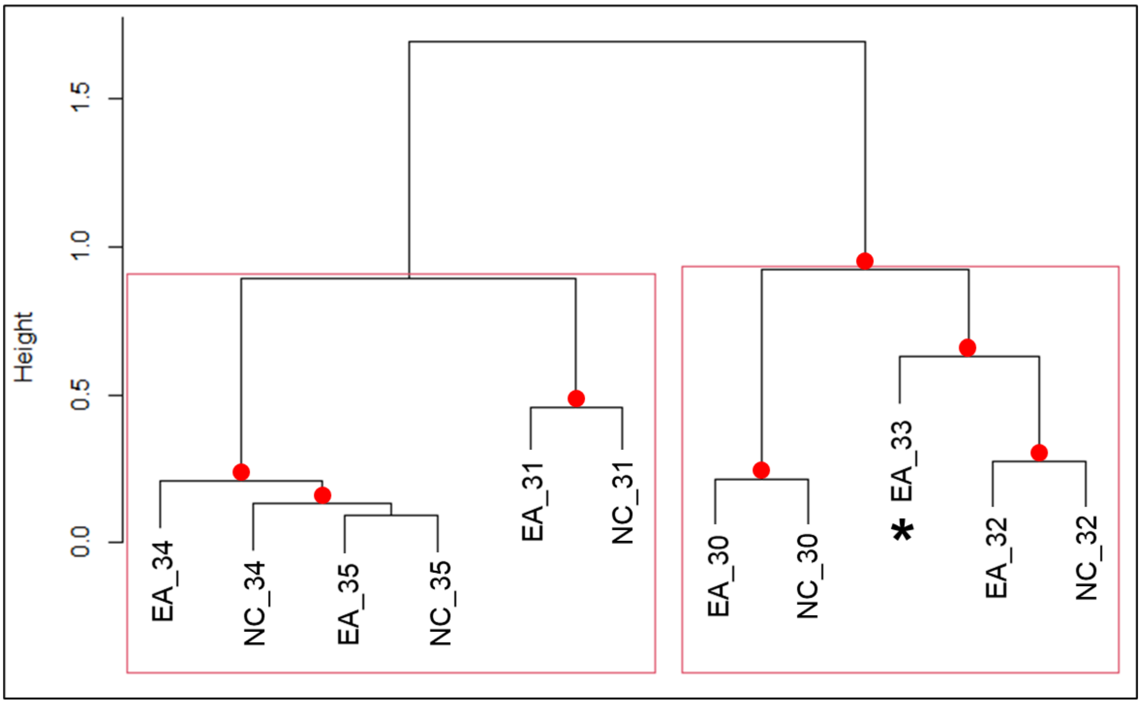
Height
0.0
0.5
1.0
1.5
EA_34
NC_34
EA_35
NC_35
EA_31
NC_31
EA_30
NC_30
* EA_33
EA_32
NC_32

d)

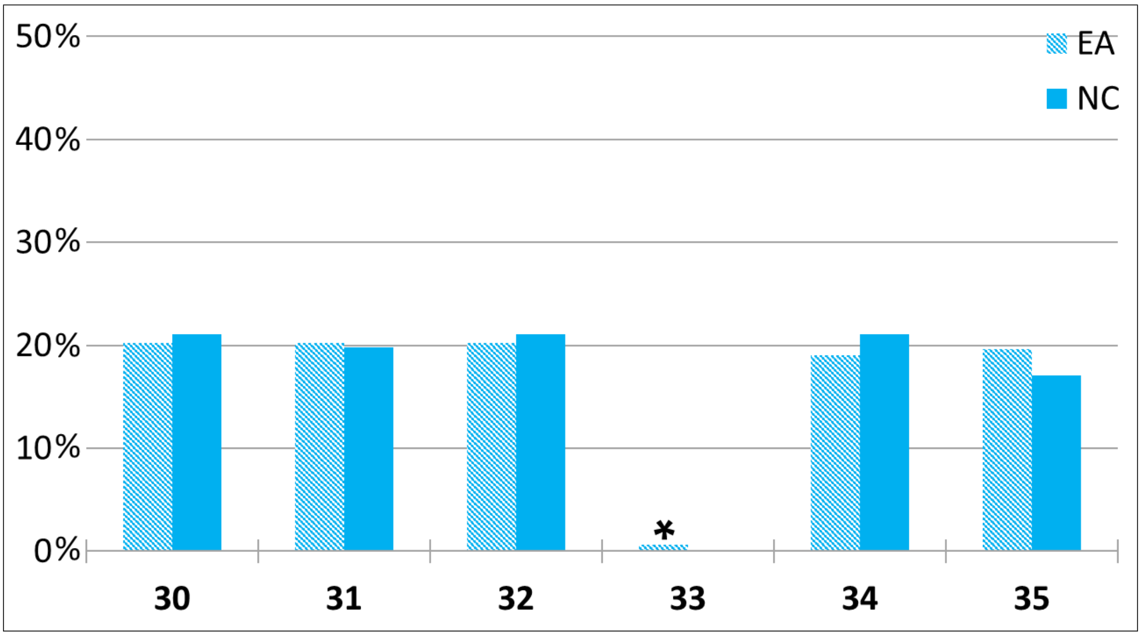
50%
40%
30%
20%
10%
0%
EA
NC
*
30
31
32
33
34
35

Figure S6a-d The Orange song type from a) east Australia 2014 and b) New Caledonia 2015

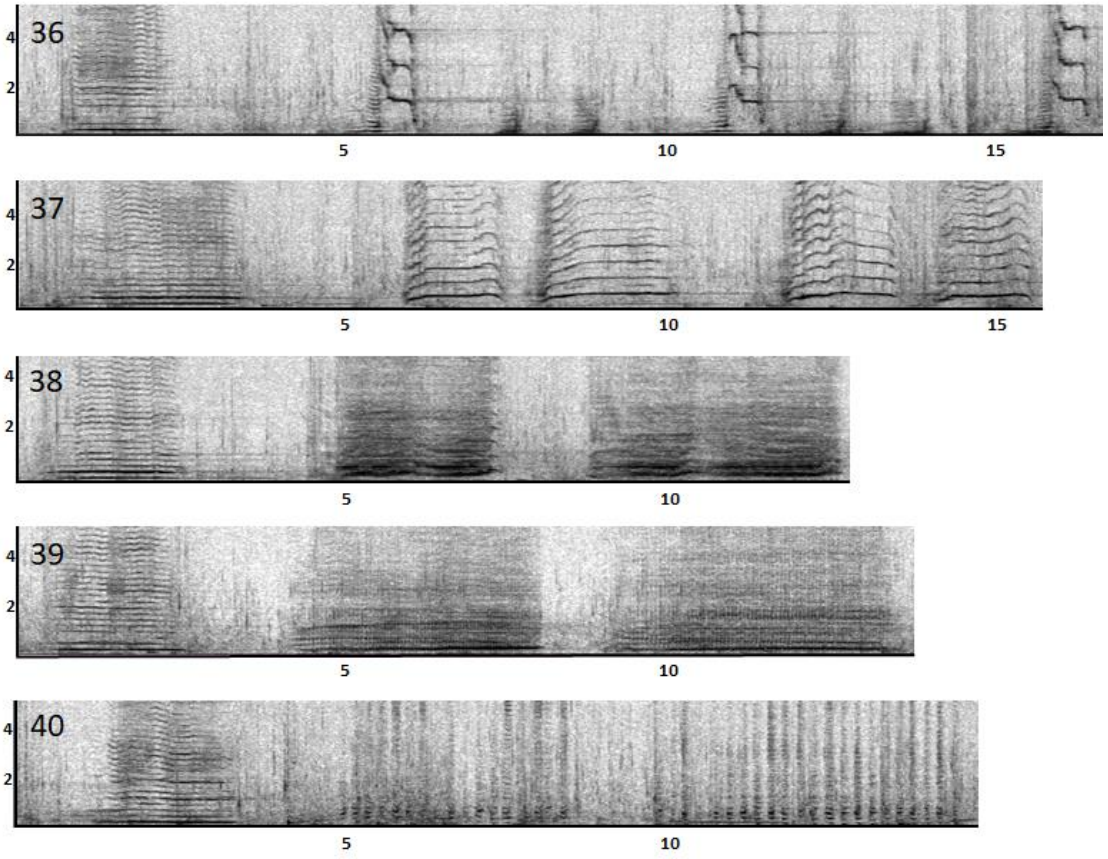

a)

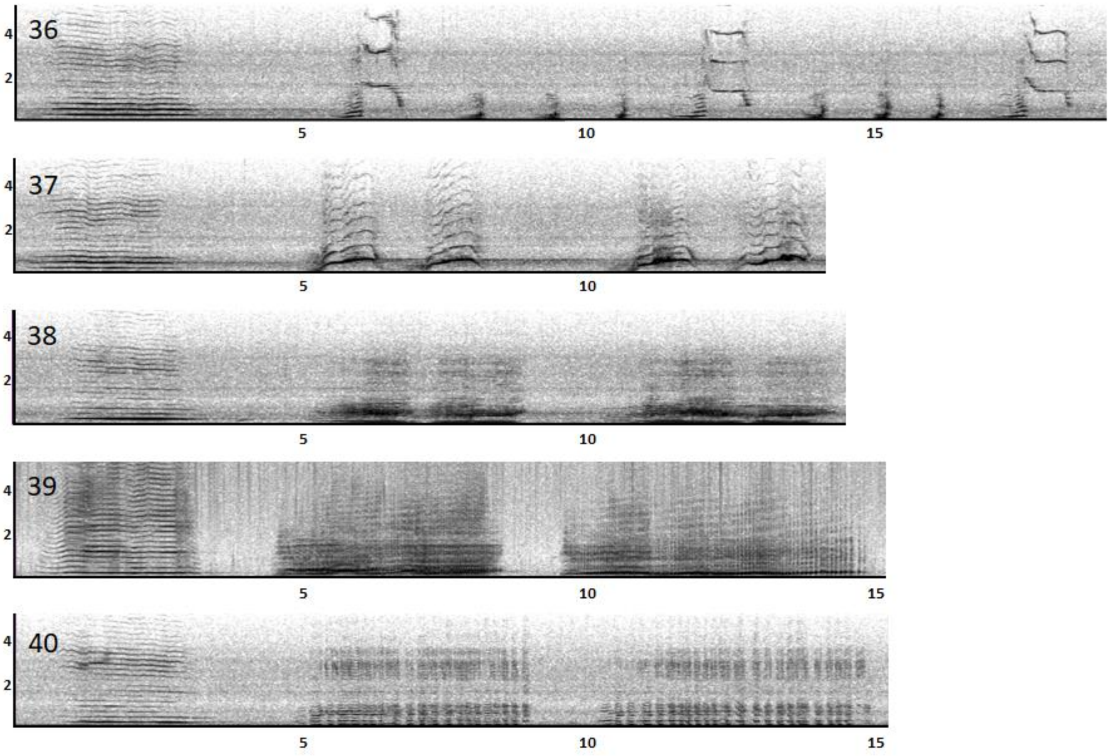

b)

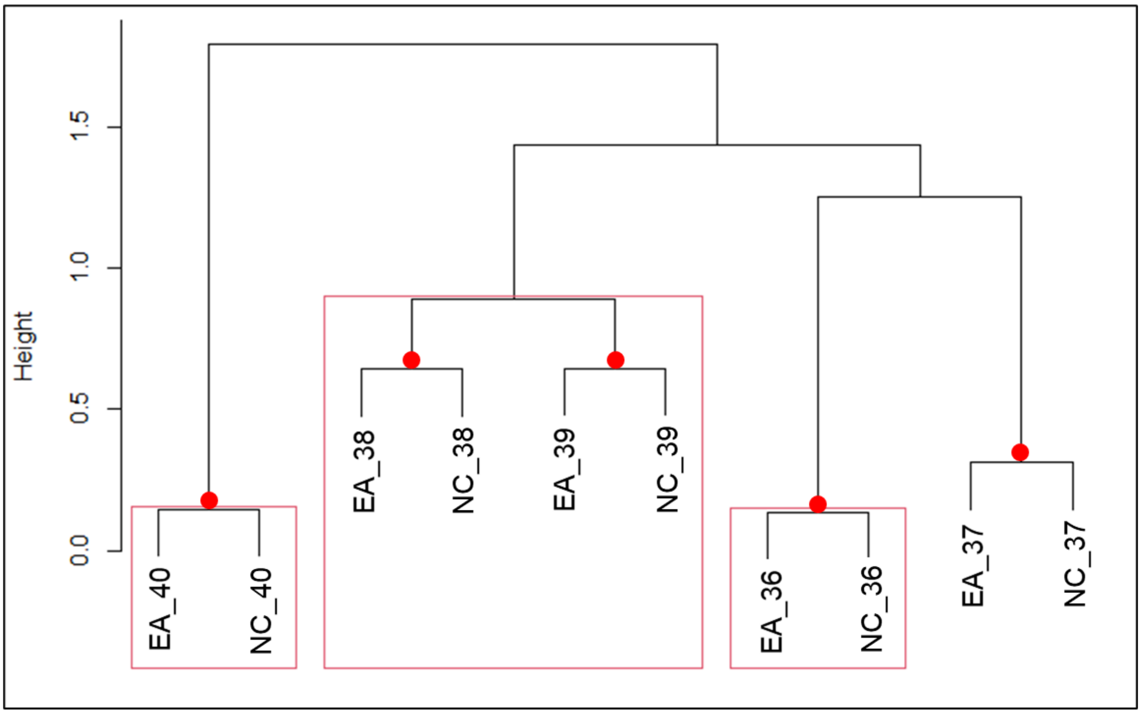
Height
1.5
1.0
0.5
0.0
EA_40
NC_40
EA_38
NC_38
EA_39
NC_39
EA_36
NC_36
EA_37
NC_37
c)

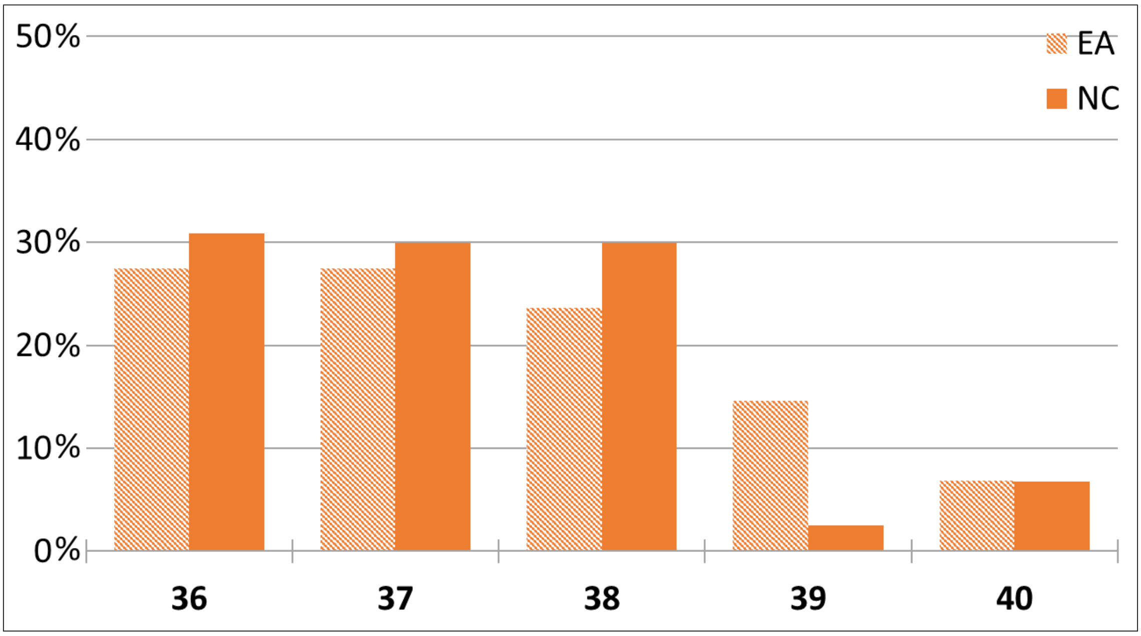
50%
40%
30%
20%
10%
0%
EA
NC
36
37
38
39
40
d)

Supplementary Figures S7 Dendrogram showing distinct song types across populations using theme sequences and an unweighted LSI. Bootstrapped (1000 times) average linkage hierarchical cluster analysis was used based on similarity matrices of set median unit sequences of themes. The height (y-axis) of two branches reflects the LSI distance between them. Red dots indicate divisions with an approximately unbiased p-value greater than 95% and are thus strongly supported by the data, with red boxes around the resulting major branches. Song types are labelled by population (EA or NC) and song type.

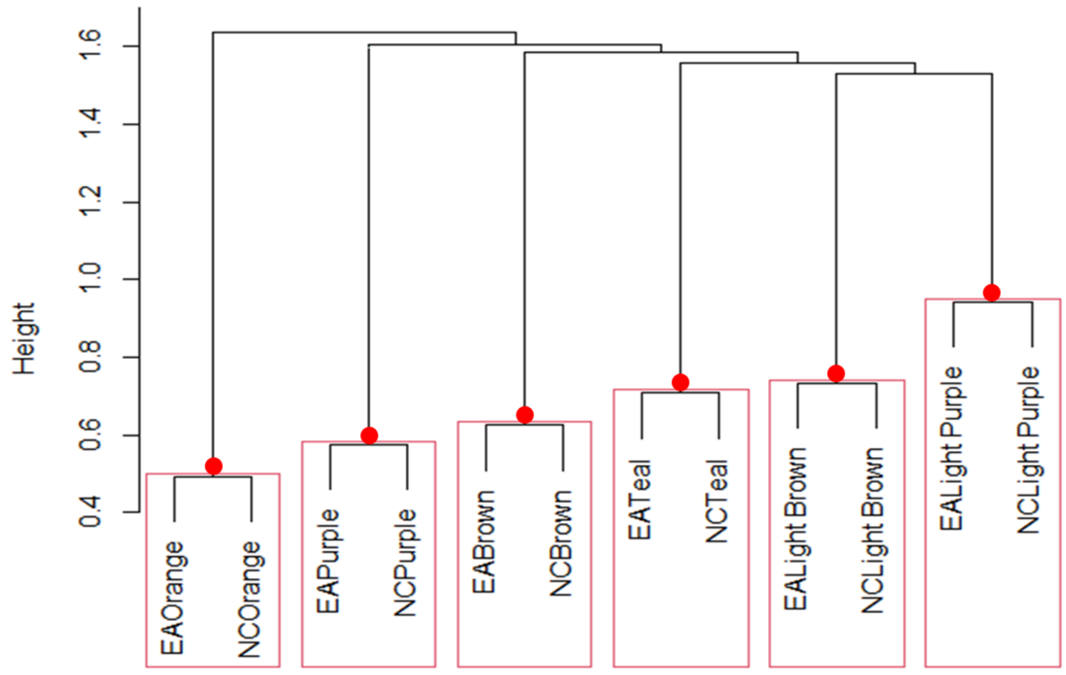

Supplementary Figures S8 Dendrogram showing full results for distinct song types across populations using theme sequences and an unweighted LSI. The height (y-axis) of two branches reflects the LSI distance between them. Each song type is represented on its own branch and colored correspondingly (Purple, Light Purple, Brown, Light Brown, Teal, Orange). Each individual song cycle is labelled by population (EA or NC), song type, and song cycle number (1-36).
